# Statins reduce new-onset atrial fibrillation after acute myocardial infarction: A nationwide study

**DOI:** 10.1097/MD.0000000000018517

**Published:** 2020-01-10

**Authors:** Chien-Hao Tseng, Wen-Jung Chung, Chen-Yu Li, Tzu-Hsien Tsai, Chien-Ho Lee, Shu-Kai Hsueh, Chia-Chen Wu, Cheng-I Cheng

**Affiliations:** a Division of Cardiology, Department of Internal Medicine, Chang Gung Memorial Hospital Kaohsiung Branch, Kaohsiung; b Clinical Informatics and Medical Statistics Research Center, Taiwan, ROC; c Foreign Language and International Trade School, Wenzhou Business College, Wenzhou, China; d Division of Cardiothoracic and Vascular Surgery, Chang Gung Memorial Hospital Kaohsiung Branch, Kaohsiung; e Chang Gung University College of Medicine, Guishan District, Taoyuan City, Taiwan, ROC.

**Keywords:** acute myocardial infarction, atrial fibrillation, statin

## Abstract

Atrial fibrillation (AF) is an important complication of acute myocardial infarction (AMI). The association between AF and serum lipid profile is unclear and statin use for lowering the incidence of new-onset AF remains controversial. The objective of this study was to investigate whether statins confer a beneficial effect on AF after AMI.

Data available in the Taiwan National Health Insurance Research Database on 32886 AMI patients between 2008 and 2011 were retrospectively analyzed. Total 27553 (83.8%) had complete 1-yr follow-up data. Cardiovascular outcomes were analyzed based on the baseline characteristics and AF type (existing, new-onset, or non-AF). AF groups had significantly higher incidence of heart failure (HF), stroke, all-cause death, and major adverse cardiac and cerebrovascular event (MACCE) after index AMI (all *P* < .05). In contrast, myocardial re-infarction (re-MI) was not significantly different among the three groups (*P* = .95). Statin use tended to be associated with lower risk of new-onset AF after AMI (HR: 0.935; 95% confidence interval (CI): 0.877–0.998; *P* = .0427).

Existing AF and new-onset AF subgroups had similar cardiovascular outcomes after AMI and were both inferior to the non-AF group. Statin tended to reduce new-onset AF after AMI.

## 1 Introduction

Acute myocardial infarction (AMI) is the leading cause of death in the world, while atrial fibrillation (AF) is the most common and clinically significant cardiac arrhythmia that is associated with higher morbidity and mortality.^[1,2]^ Additionally, AF is a common complication of AMI and is associated with poor outcomes, especially major adverse cardiac and cerebrovascular event (MACCE) in AMI patients. Many risk factors for AF and AMI have been identified and are being used to clinically stratify patients to investigate adequate management of these specific risk factors. Previous reports show that statin use is associated with lower incidence of new-onset AF.^[3,4]^ Thus, the objective of this analysis was to assess the correlation between AF and AMI and to investigate the effects of a lipid lowering agent on MACCE in AMI patients with and without AF.

## 2 Methods

### 2.1 Database

The National Health Insurance of Taiwan, launched in March 1995, is a single-payer health care program that provides universal access to inpatient care, outpatient care, dental care, and prescription medications for over 99.9% of Taiwanese residents. Besides, the insurance system is also contracted with 97% of the hospitals and clinics in Taiwan.^[5]^ Data from the National Health Insurance Research Database (NHIRD) was used in this study and included all inpatient and outpatient medical claims between January 1, 2008 and December 31, 2011.^[6]^ The Institutional Review Board of the Chang Gung Memorial Hospital approved this study (102–3429B). The need for informed consent was waived as all records accessed from the NHIRD were de-identified before analysis.

### 2.2 Study design and population

The selection criteria for potential inclusion in this retrospective cohort study were adult patients who were 18 years or older and admitted to any hospital, including regional hospital and medical center through the Emergency Department (ED) with an established diagnosis of AMI. In the database, AMI admission was defined according to ICD9-CM (International classification of Diseases, Ninth Revision, Clinical Modification) criteria as hospitalization with a primary diagnosis code of 410.x. The database contained some cases with an incorrect diagnosis of AMI due to ICD-9 coding errors, patients with out-of-hospital cardiac arrest, or patients diagnosed with AMI without hospitalization. To prevent the inclusion of such cases in the current analysis, we focused on non-fatal AMI patients hospitalized via ED and discharged alive. The following exclusion criteria were applied:

(1) patients under 18 years of age,
(2) patient death in the ED,
(3) patients admitted for other etiologies but with subsequent in-hospital AMI, and
(4) patients with thyroid disease (ICD-9 codes 193, 240.9, 242.9, 244.9, and 648.13).

**Figure 1:**
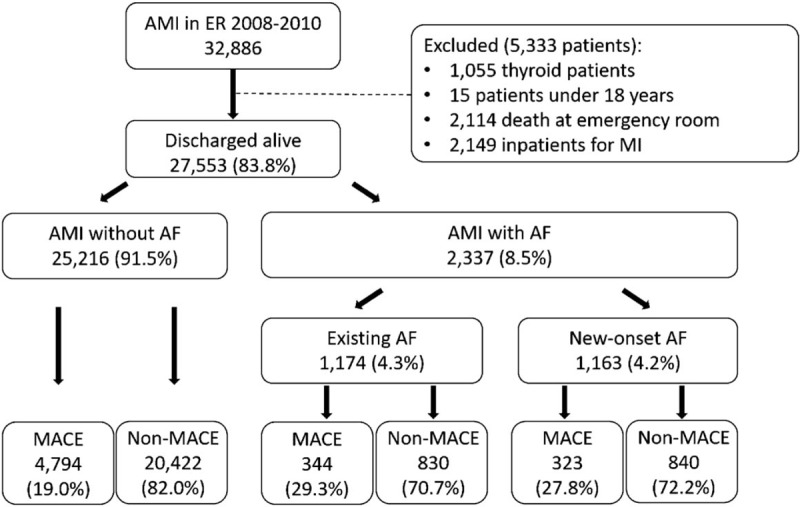
Patient Selection in NHIRD. The study population of patients who treated as acute myocardial infarction was selected from 2008 to 2010 in the NHIRD, included 32886 AMI patients. (AF = atrial fibrillation, AMI = acute myocardial infarction, MACE = Major adverse cardiovascular event, NHIRD = National Health Insurance Research Database).

Figure 1 presents a flowchart depicting AMI patient identification from NHIRD, data management, and study design.

### 2.3 Study groups

Patient data collected from NHIRD included age, gender, comorbidities, and treatment information (Tables 1 and 2). For each patient, data on comorbidities were retrieved from both inpatient and outpatient charts for the period of 1 year before and during the index AMI event. Selected patients were initially divided into 2 groups as AMI with AF or AMI without AF (ICD-9 code 427.31). Next, based on index event time, patients with AF were categorized further into 2 groups as AF existing before the index event or new-onset AF occurring during the hospitalization period of the index event.

**Table 1:**
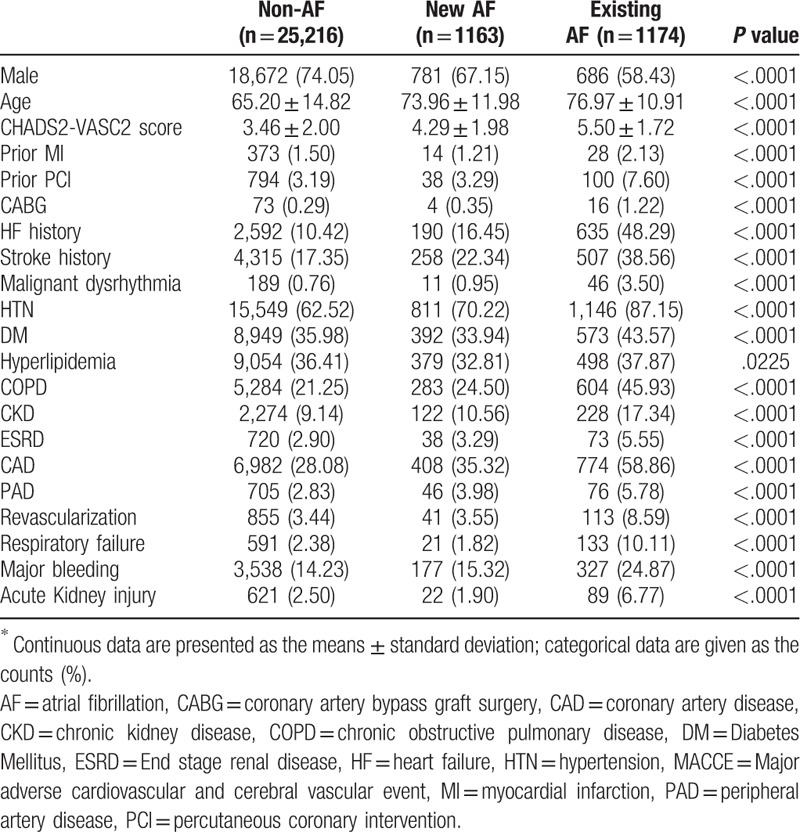
Baseline characteristics between discharge diagnosis with non-AF, new AF, and existing AF among AMI patients^∗^.

**Table 2:**
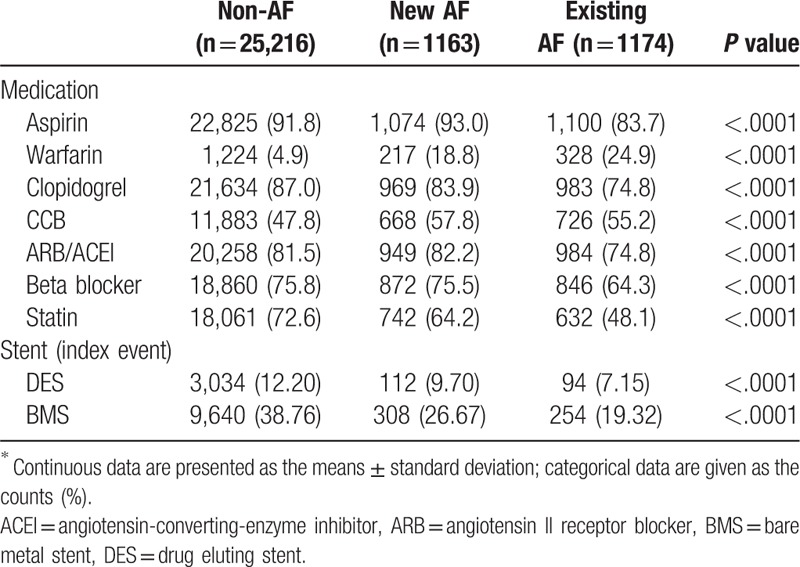
Cardiovascular medications and stent strategy during hospitalization^∗^.

### 2.4 Outcomes

The multiple primary endpoint included recurrent MI, admission for HF (ICD-9 code 428.0 & 428.1), stroke (cerebrovascular accident, CVA; ICD-9 code 430–437), all-cause death, and MACCE which consisted of recurrent MI, admission for (HF), stroke, and death. To identify mortality-related cardiovascular events, we used data from the death certificates in the NHIRD and screened for diagnostic codes associated with cardiovascular events. The CHA2DS2-VASc score was calculated by assigning 1 point each for history of congestive heart failure or left ventricular dysfunction, hypertension, age between 65 and 74 years, diabetes mellitus, vascular disease (myocardial infarction or peripheral artery disease), female sex and 2 points each for age >75 years, history of a stroke or transient ischemic attack.^[7]^

### 2.5 Statistical analysis

Continuous variables were expressed as mean ± standard deviation and were compared using one-way ANOVA. Categorical variables were expressed as numbers and percentages and were compared using the Chi-square test. The Cox proportional hazard model was used to identify associations between multiple independent predictors and 1-year MACCE occurrence and is expressed as hazard ratio and 95% confidence interval (CI). The Kaplan-Meier survival curve was used to estimate overall MACCE and primary outcome; the log-rank test was used to compare groups and treatments. All statistical analyses were performed using SAS statistical software, version 9.4 (SAS Institute Inc., Cary, NC), and *P* value < .05 was considered statistically significant.

## 3 Results

The study included 32,886 AMI patients during the years 2008 to 2010, for whom follow-up data for the first year after hospital discharge of index AMI event was available. Data from 5333 patients (16.2%) were excluded due to several factors such as thyroid disease, age below 18 years (n = 15, 0.05%), death in the emergency room, and inpatient MI. Thus, records of 27553 (83.8%) AMI patients who were discharged alive and had completed 1-year follow-up was used for analysis; totally, AF groups comprised 1174 (4.3%) patients with existing AF and 1163 (4.2%) patients with new-onset AF during hospitalization.

The demographic characteristics of the study population (Table 1) show that mean CHA_2_DS_2_-Vasc scores were significantly different among the 3 groups with higher scores seen in AMI patients with AF (non-AF: 3.46 ± 2.00, new-onset AF: 4.29 ± 1.98, existing AF: 5.50 ± 1.72, respectively, *P* < .001). The AF groups had older patients and a higher percentage of heart failure and stroke; these were highest in the existing AF group. Compared to the baseline, among the three groups, the existing AF group exhibited the highest incidence rates of prior MI and CABG, and a higher incidence rate of HF, stroke, hypertension (HTN), diabetes mellitus (DM), chronic kidney disease (CKD), chronic obstructive pulmonary disease (COPD), coronary artery disease (CAD), respiratory failure, major bleeding, acute kidney injury, and hyperlipidemia (all *P* value < .05).

The existing AF group also had lowest percentage of treatment with Aspirin, Clopidogrel, Statin, ARB/ACEI, and beta-blockers (all *P* value < .05) at medical discharge from the hospital (Table 2).

To investigate the impact of baseline characteristics associated with new-onset AF after AMI, the multivariate Cox proportional hazards model was used. These results demonstrate that age, HF, DM, HTN, stroke, and CKD were all significant risk factors that were independently associated with the increased incidence of new-onset AF after AMI (Table 3, all *P* value < .05). Moreover, prescription of ARB/ACEI and beta-blockers at medical discharge were both associated with higher risk of new-onset AF after AMI; interestingly, statin use tended to be associated with lower risk of new-onset AF after AMI (HR: 0.935; 95% CI: 0.877–0.998; *P* = .0427).

**Table 3:**
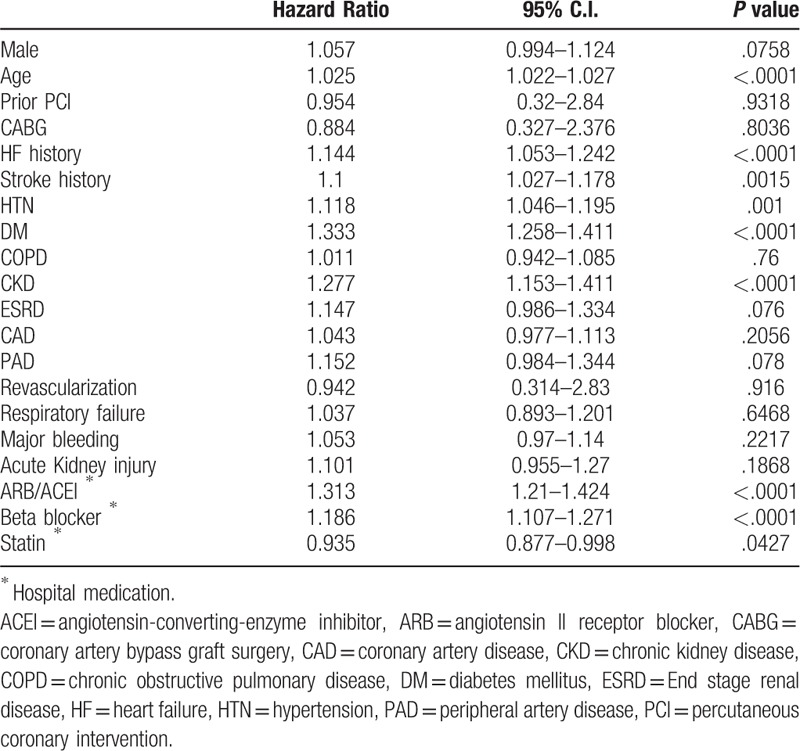
Multivariate analysis for independent predictor of new-onset AF after AMI.

To evaluate the impact of AF on the incidence of cardiovascular events and MACCE, we stratified AMI patients into three groups based on AF presence as new-onset, existing, and non-AF, and compared 1-year MACCE incidence. Survival estimates in AMI patients showed that the non-AF groups had significantly lower incidence of HF, stroke, death, and MACCE after index AMI (Fig. 2  B–E) and that the new-onset AF group had the worst outcome regarding heart failure and MACCE (Fig. 2  B and E) in the first 3 months after the index AMI event. In contrast, the outcome of myocardial re-infarction (re-MI) was not significantly different among the 3 groups (Fig. 2  A).

**Figure 2:**
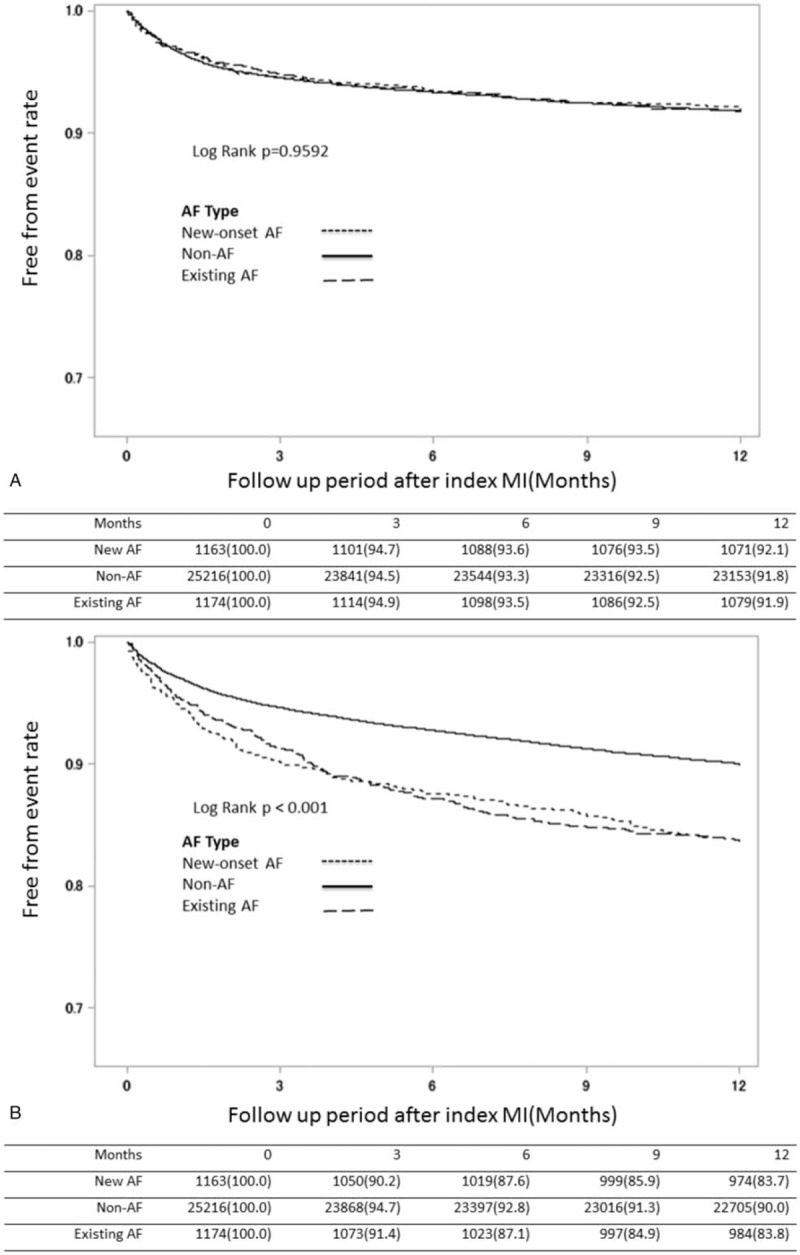
Kaplan–Meier survival estimates after index MI among groups of different AF patterns. Kaplan-Meier survival estimates of entire patient cohort stratified by AF after index MI. The MI-free (A), heart failure-free (B), stroke-free (C), overall (D) and MACCE-free (E) survival estimates for the patient cohort are grouped by AF pattern which is represented by lines as denoted in the panel. (AF = atrial fibrillation, MACCE = major adverse cardiac and cerebral vascular events, MI = myocardial infarction).

**Figure 2 (Continued):**
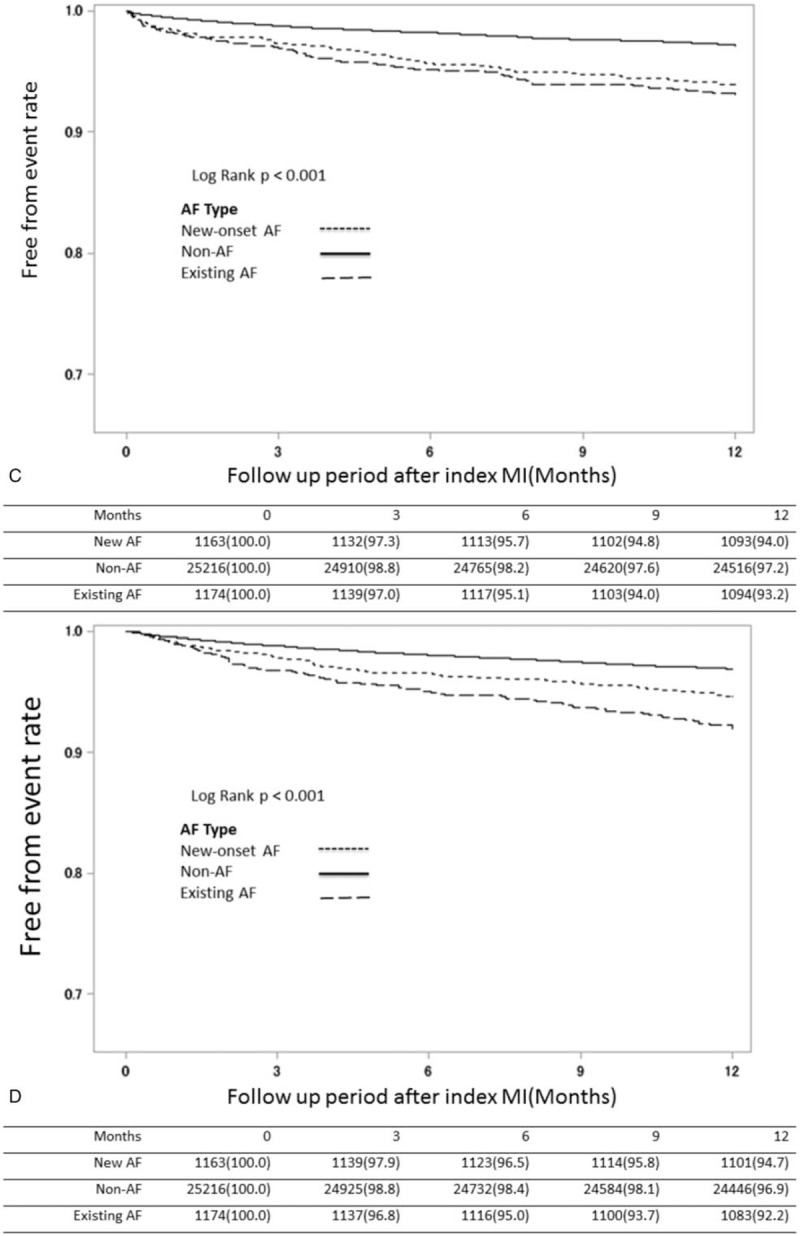
Kaplan–Meier survival estimates after index MI among groups of different AF patterns. Kaplan-Meier survival estimates of entire patient cohort stratified by AF after index MI. The MI-free (A), heart failure-free (B), stroke-free (C), overall (D) and MACCE-free (E) survival estimates for the patient cohort are grouped by AF pattern which is represented by lines as denoted in the panel. (AF = atrial fibrillation, MACCE = major adverse cardiac and cerebral vascular events, MI = myocardial infarction).

**Figure 2 (Continued):**
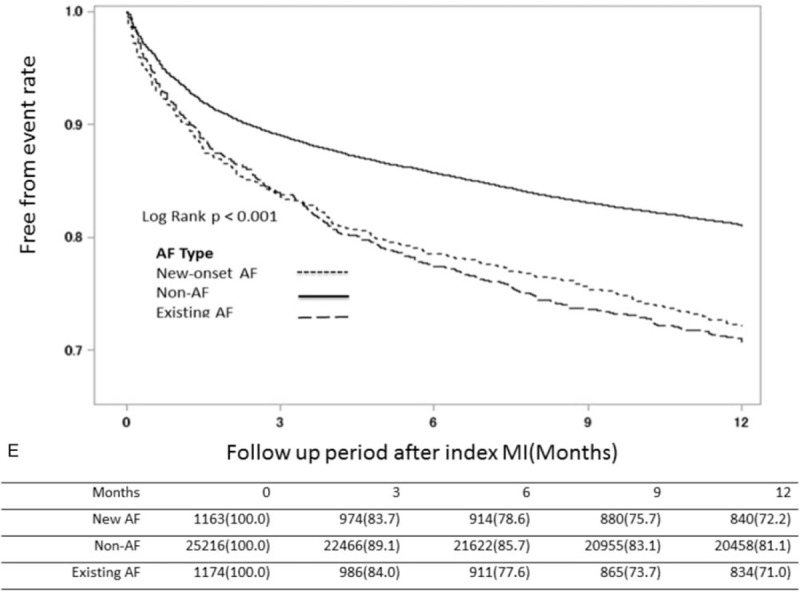
Kaplan–Meier survival estimates after index MI among groups of different AF patterns. Kaplan-Meier survival estimates of entire patient cohort stratified by AF after index MI. The MI-free (A), heart failure-free (B), stroke-free (C), overall (D) and MACCE-free (E) survival estimates for the patient cohort are grouped by AF pattern which is represented by lines as denoted in the panel. (AF = atrial fibrillation, MACCE = major adverse cardiac and cerebral vascular events, MI = myocardial infarction).

The multivariate Cox model for mortality and MACCE at 1 year was used to study the relative risk of 1-year death and 1-year MACCE in AMI patients. Table 4 indicates that AMI patient death within 1 year after the index AMI event were significantly associated with age, HF history, stroke history, HTN, DM, CKD, ESRD, prescription of Warfarin, Clopidogrel, ARB, and beta-blockers at medical discharge, and new-onset AF (all *P* < .05) but Percutaneous coronary intervention with the implantation of DES (HR:0.745, 95% C.I.: 0.672–0.826, *P* < .0001) and prescription of statin at discharge reduced death within 1 year after the index AMI event (HR:0.923, 95% C.I.:0.865–0.984, *P* = .0149).

**Table 4:**
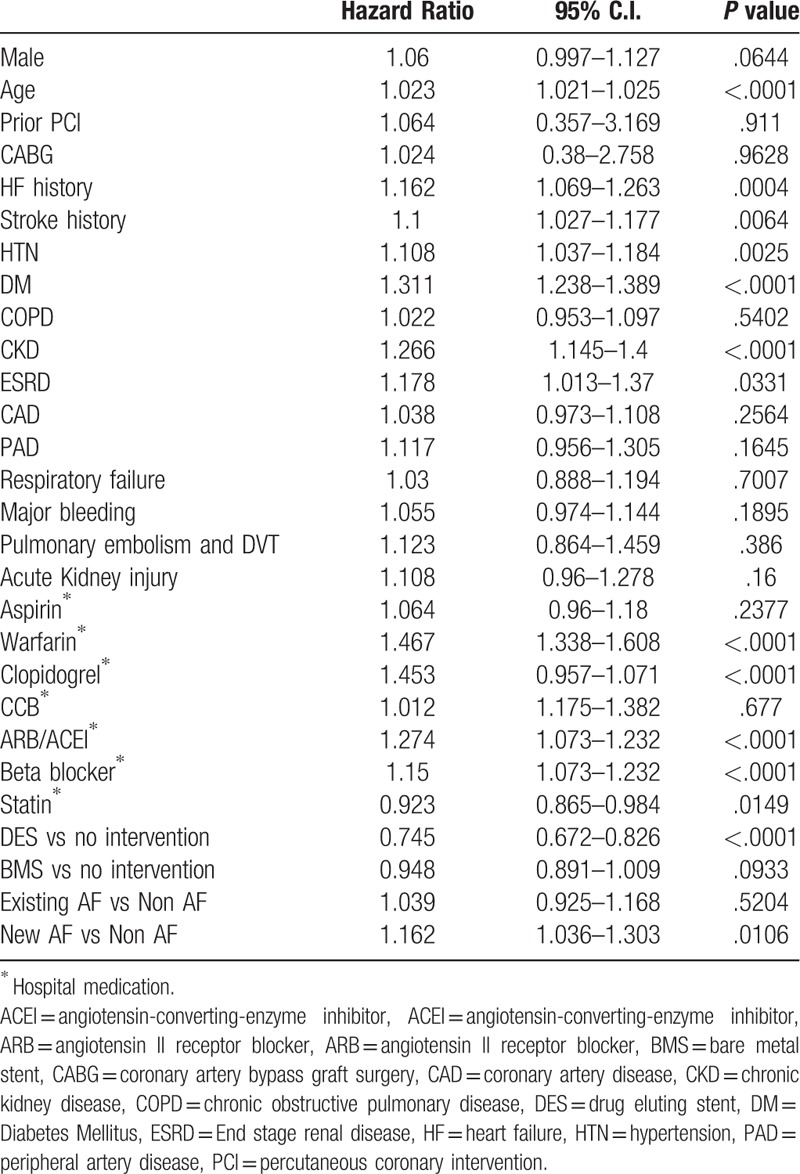
Multivariate cox model for independent predictor of death at 1 year.

Table 5 indicates that AMI patients suffer from MACCE within 1 year were significantly associative with age, HF history, stroke history, HTN, DM, CKD prescription of Warfarin, Clopidogrel, ARB, beta-blockers at medical discharge and new-onset AF (all *P* < .05) but DES implantation (HR:0.728, 95% C.I.: 0.657–0.807, *P* < .0001) and prescription of statin at discharge reduced death within 1 year after the index AMI event (HR:0.936, 95% C.I.: 0.877–0.999, *P* = .0463).

**Table 5:**
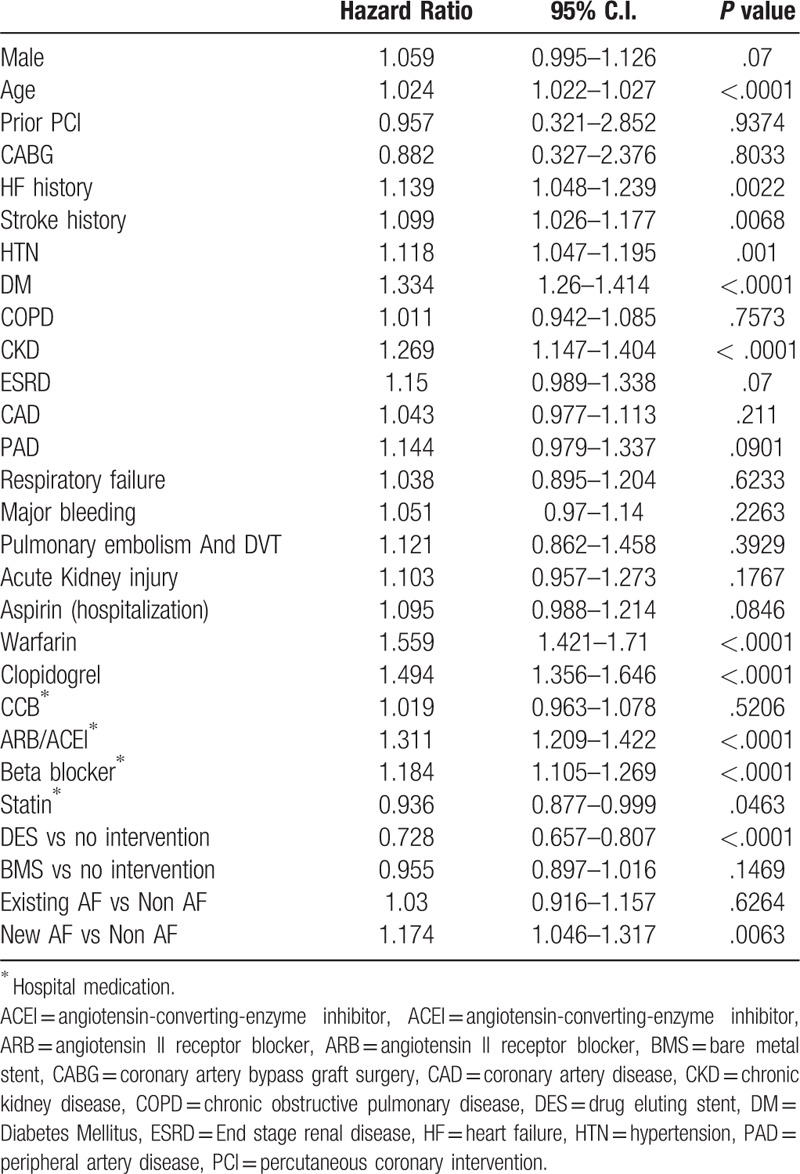
Multivariate cox model for independent predictor of Major adverse cardiovascular and cerebral vascular event at 1 year.

**Figure 3:**
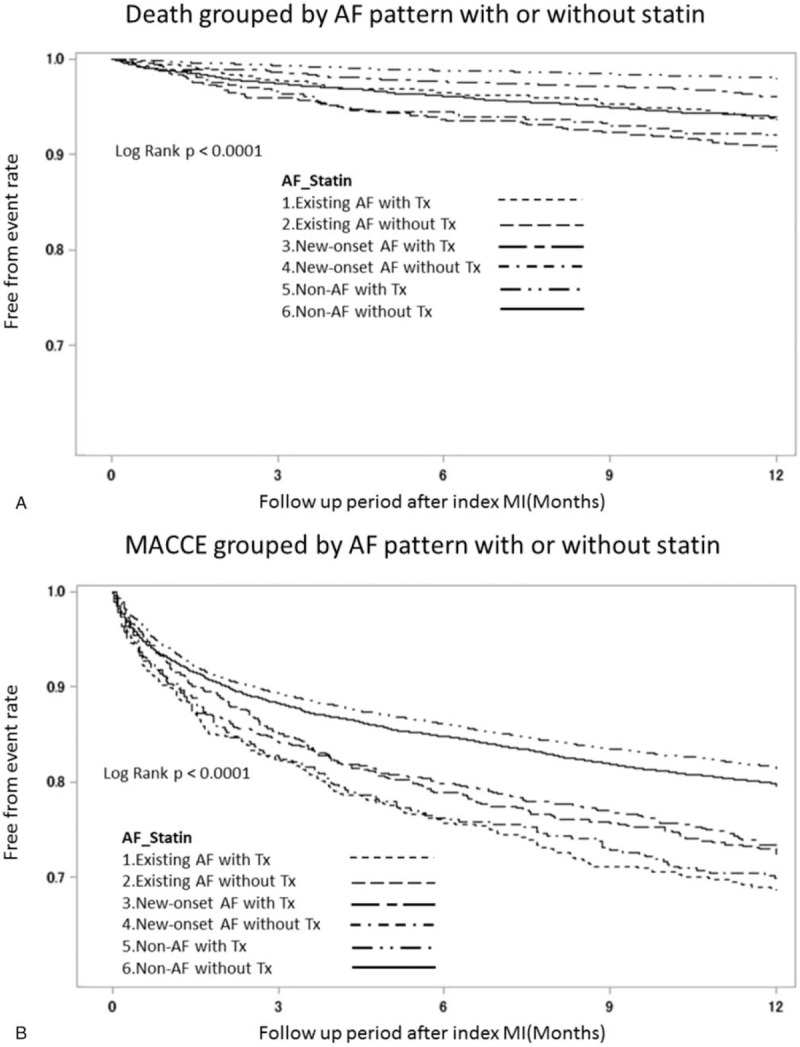
Kaplan–Meier estimates grouped by statin treatment and AF pattern. Kaplan-Meier survival estimates of AF patient after MI stratified by statin treatment and AF pattern after index MI. The overall (A) and MACCE-free (B) survival estimates for the patient cohort are grouped by AF pattern and statin treatment which is represented by lines as denoted in the panel. (AF = atrial fibrillation, MACCE = major adverse cardiac and cerebral vascular events, MI = myocardial infarction).

Next, we also examined the clinical effect of treatment with or without lipid lowering agents (statins) in patients with non-AF, existing AF, and new-onset AF with respect to HF, recurrent MI, stroke, death and MACCE. As seen in Figure 3A and B, better outcomes regarding mortality were seen in the treatment group, irrespective of AF. Specifically, better outcomes were observed in the new-onset-AF-with-Statin sub-group than the non-AF without Statin sub-group; worse outcomes were seen in the new-onset AF without Statin group than the existing AF with Statin group. In contrast, MACCE outcomes were worse in the existing-AF-with-Statin sub-group compared to the other groups (Fig. 3A and B).

## 4 Discussion

We have investigated data stored in the Taiwanese national database to retrospectively analyze the influence of multiple independent predictors on the association of AF and AMI patients. Our previous study about AMI patient population have reported a significantly higher incidence of hyperlipidemia and statin use in AMI patients who do not develop AF (non-AF group).^[8]^ The current study makes further data analysis. Although the heterogeneity of the patient populations may be a potential confounding factor, multivariate analysis demonstrates that the new-onset AF is still a significant predictor for MACCE in this real-world data.

About our study, it shows some interesting findings. First, statin use tended to be associated with lower risk of new-onset AF after AMI (HR: 0.935; 95% CI: 0.877–0.998; *P* = .0427). Second, the new-onset AF group had the worst outcomes with respect to HF and MACCE (Fig. 2  B and E) within the first 3 months after the index AMI event. Third, the new-onset-AF-with-Statin sub-group demonstrated favorable outcomes with respect to mortality compared to the non-AF-without-Statin sub-group; in contrast, the new-onset-AF without-Statin sub-group had worse mortality outcomes than the existing-AF-with-Statin sub-group. Last, the existing-AF-with-Statin sub-group seemed to have the worst MACCE outcome than other sub-groups and patients under therapy had better outcome than those without therapy, even in groups with high CHA_2_DS_2_-Vasc scores.

Among the three groups, the existing-AF group had higher mean CHA_2_DS_2_-Vasc scores (existing AF: 5.50 ± 1.72, new-onset AF: 4.29 ± 1.98, non-AF: 3.46 ± 2.00, respectively, *P* < .001), greater age, and comorbidities [including higher incidence rates of HF, stroke, hypertension (HTN), DM, CKD, COPD, CAD, respiratory failure, major bleeding and acute kidney injury], all of which can contribute to poor MACCE outcome. Previous large-scale studies have investigated the effects of statins on new-onset atrial fibrillation (AF) in CKD or dialysis patients but rarely in AMI patients^[9]^; thus, this study focuses on the effects of statin prescription after AMI.

### 4.1 AF, mortality, and MACCE

Available literature^[10]^ states that the incidence of AF as an AMI complication is as high as 21%. AF is the most common arrhythmia and is associated with increased risk of short-term and long-term mortality.^[11,12]^ Compared to AMI patients without AF, those with AF have a higher risk of in-hospital and long-term MACCE incidence. A major finding of our study was that the relative risk of MACCE within 1 year after AMI was significantly higher in the AF group than in the non-AF group, especially new-onset AF within the first 3 months. AF indicates poor MACCE outcome of AMI patient follow-up at 1 year.

### 4.2 AF and the pleiotropic effect of statin

Statins act mainly by lowering serum cholesterol through the inhibition of cholesterol synthesis in the liver, which results in the upregulation of low-density lipoprotein (LDL) receptors in the liver and the removal of low-density lipoprotein-cholesterol (LDL-C). Previous studies have suggested that statins may have effects independent of LDL-C lowering,^[13]^ termed pleiotropic effects.^[2,14]^ These pleiotropic effects include the inhibition of isoprenoid intermediates by statins and has effects on the small guanosine triphosphate binding proteins Rho, which in turn effects on nicotinamide adenine dinucleotide phosphate (NADPH) oxidases.^[15,16]^ Thus, there are changes in endothelial nitric oxide synthase expression, atherosclerotic plaque stability, pro-inflammatory cytokines and reactive oxygen species production. Furthermore, AF has also been associated with increased atrial oxidative stress and NADPH oxidase activity.^[17]^ Statin treatment reduces NADPH oxidase activity pro-and inflammatory markers, such as CRP as well as the incidence of AF in a mouse model.^[2,18,19]^

Previous studies did not ascertain the causal link between elevated LDL-C level and atrial fibrillation (AF).^[20,21]^ WOSCOPS study showed a non-statistically significant trend on inflammation regulation of statin.^[22]^

On the contrary, Atorvastatin can significantly regulate inflammation based on previous Li et al study.^[23]^ In addition, the basic science data of the post-hoc analysis of JUPITER study showed a 27% reduced risk of developing AF, and increased AF incidence was associated with increased CRP, which suggested that the anti-inflammatory effects of statins may reduce AF.^[24]^ Furthermore, meta-analysis of statin also showed conflicting results regarding AF prevention.^[4,25,26]^ For the effect of statins on the prevention of AF recurrence in patients with known AF, a small randomized controlled trial enrolled patients with an elevated CRP and a previous diagnosis of paroxysmal AF; it found a 65% reduction of AF recurrence and a significant reduction in CRP after 6 months of treatment with atorvastatin.^[27]^ Thus, the benefit of statins to prevent AF is not universally seen. Further large-scale randomized controlled trials are necessary to prove the causality.

### 4.3 Upstream therapy of AF

The RealiseAF study^[28,29]^ demonstrated that 5% of the total population had AF alone; this is a diagnosis of exclusion when no other comorbid CV conditions are manifest. However, it must be noted that most patients with AF generally have multiple cardiovascular (CV) comorbidities.^[30]^ The correlation between hyperlipidemia and AF is controversial.^[31]^ The results of the Atherosclerosis Risk in Communities study^[32]^ revealed that high levels of high-density lipoprotein cholesterol were associated with lower AF risk (hazard ratio 0.64, 95% CI 0.48 to 0.87 in those with levels ≥60 mg/dL versus < 40 mg/dL), whereas high triglycerides were associated with higher risk of AF (hazard ratio 1.60, 95% CI 1.25 to 2.05 in those with levels ≥200 mg/dL versus < 150 mg/dL). Besides, total cholesterol and low-density lipoprotein cholesterol were not associated with the risk of AF. In contrast, both the Multi-Ethnic Study of Atherosclerosis (MESA) and the Framingham Heart Study (FHS)^[33]^ showed that HDL-C and triglycerides (not total cholesterol or LDL-C) levels were associated with increased risk of new-onset AF in women but not in men (in women, hazard ratio 2.86; 95% confidence interval: 1.49–5.50; in men, HR, 1.35; 95%CI: 0.77–2.38). Thus, the association between serum lipid profile to new-onset AF remains unclear.

In our study, we compared patients in three groups and demonstrate that patients in the non-AF group had significantly higher incidence of hyperlipidemia and statin use compared to those in the AF group. Furthermore, the New-onset-AF-with-Statin sub-group had lower mortality than the non-AF-without-Statin sub-group and that the new-onset-AF-without-Statin sub-group had worse mortality than the existing- AF-with-Statin sub-group (Fig. 3A and B). Thus, even though previous studies have shown inconsistent evidence for reduction of the incidence of new-onset AF in acute coronary syndrome (ACS) patients due to statin use,^[34]^ we consider that statin therapy for AF, especially new-onset AF, may reduce the incidence of HF and MACCE after AMI.

Upstream therapy is an alternative choice to ion-channel-based antiarrhythmic drugs that modify the atrial substrate or target-specific mechanisms for AF treatment. These drugs include ACEIs, ARBs, statins, or omega-3 polyunsaturated fatty acids. Although animal studies have provided considerable evidence for the benefits of upstream therapy, there is insufficient evidence from human studies to recommend the widespread use of these agents for AF prevention.

In our study, prescription of ARB/ACEI and beta-blockers were both associated with higher risk of new-onset AF after AMI. This observation has to be considered in light of the fact that no Taiwanese guidelines were available during 2008 to 2011 for AMI or AF management. Furthermore, the underlying cause of this observation appears to be that the AF group had greater incidence of comorbidities (HF, hypertension, CAD), which may need ARB/ACEI and beta-blockers for management. In fact, these regimens confirm to recommendations of the 2012 TSOC HF Guideline and the 2012 TSOC AMI Guideline.^[35,36]^ Furthermore, previous literature reviews and TSOC AF Guideline^[37]^ suggest that ACEI may reduce the incidence of AF in the chronic phase among patients with left ventricular dysfunction secondary to an AMI.^[38]^ Specifically, the CAPRICORN study showed that Carvedilol also has a beneficial effect in suppressing AF and atrial flutter in the AMI patient population.^[39]^

### 4.4 Clinical implication

Previous studies have indicated that statin therapy might be effective in protecting against AF after ACS^[40]^ and CAD,^[41]^ but the strongest evidence for AF prevention by statins is seen in post-cardiac surgery patients.^[42]^ The current guideline suggests that AF requires anticoagulant therapy for stroke prevention, although anticoagulant combined with dual anti-platelet therapy after percutaneous coronary intervention might increase bleeding risk.^[43,44]^ Therefore, prediction and prevention of AF as a complication of AMI is a meaningful strategy.

### 4.5 Study limitation

There are some limitations in this study. First, this is a retrospective, nonrandomized, observational data design. In addition, imbalances of baseline characteristics might have confounded the results, such as DES/BMS implantation, the use of beta-blockers, angiotensin-converting enzyme inhibitors, which could account for the MACCE outcome of CAD. Further studies may be needed to obtain stronger evidence to eliminate this bias. Second, we could not distinguish between AF and atrial flutter and the pattern of AF because of their identical ICD codes. Third, as the study population excluded patients with fatal MI, the accuracy of outcome predictions could not be evaluated in this cohort. Finally, during 2008 to 2011, as novel oral anti-coagulants (NOAC) were unavailable in Taiwan, the effects of NOAC on these outcomes in AMI patients with AF could not be evaluated. Therefore, the optimal treatment strategy in this patient population in the NOAC era may need further investigation.

## 5 Conclusion

Our study reports that as existing AF and new-onset AF patients show unfavorable outcomes after AMI; statin use tended to be associated with lower risk of new-onset AF after AMI and the patients of AMI treated with statins has lower mortality at 1 year regardless of AF pattern.

## Author contributions

**Conceptualization:** Chien-Hao Tseng, Tzu-Hsien Tsai, Chien-Ho Lee, Shu-Kai Hsueh, Chia-Chen Wu, Cheng-I Cheng.

**Data curation:** Chen-Yu Li, Tzu-Hsien Tsai, Chien-Ho Lee, Shu-Kai Hsueh, Chia-Chen Wu.

**Formal analysis:** Chen-Yu Li.

**Methodology:** Chen-Yu Li.

**Supervision:** Wen-Jung Chung, Cheng-I Cheng.

**Visualization:** Chien-Hao Tseng, Chen-Yu Li.

**Writing – original draft:** Chien-Hao Tseng, Wen-Jung Chung.

**Writing – review & editing:** Cheng-I Cheng.

Chien-Hao Tseng orcid: 0000-0003-1929-3782.
